# Coming to terms with the need for home care: a reflexive thematic analysis of older adults’ experiences in Sweden

**DOI:** 10.1080/17482631.2026.2707705

**Published:** 2026-07-24

**Authors:** Pernilla Alencar Siljehag, Åsa von Berens, Bettina Meinow, Ann Liljas, Janne Agerholm

**Affiliations:** a Stockholm Gerontology Research Center, Stockholm, Sweden; b Aging Research Center, Karolinska Institutet, Stockholm University, Stockholm, Sweden; c Department of Global Public Health, Karolinska Institutet, Stockholm, Sweden

**Keywords:** Ageing, long-term care, home care services, older people, Sweden, health services needs and demand, transition theory in nursing, qualitative research, patient acceptance of health care, life change events

## Abstract

**Background:**

Although publicly funded home care in Sweden is available to all based on municipal needs assessments, many individuals ≥65 years do not apply until their needs have become extensive. The aim of this study was to explore how older people experience the phase of life leading up to their decision to apply for home care for the first time.

**Methods:**

Individual, semi-structured interviews with 14 men and women aged 67–94 years with first-time home care in 2024 in Stockholm, Sweden, were analysed with an experiential and inductive approach to reflexive thematic analysis. Transition theory in nursing was applied as a sensitising framework.

**Results:**

We created 35 codes and the theme ‘Coming to terms with the need for home care ‒ a movement that ebbs and flows’, describing the fluctuating process of accepting increasing needs for support through home care. While some navigated this process pragmatically, for others it involved identity redefinition. Significant others sometimes became a driving force in the individual’s process of acceptance.

**Conclusion:**

These findings expand the understanding of why individuals may hesitate and delay entering home care despite having support needs and suggest that some may initiate home care use while remaining ambivalent about this decision.

## Introduction

*Yes, it is hard when drops are falling.*

*Trembling with fear, and heavy hanging,*

*cleaving to the twig, and swelling, sliding -*

*weight draws them down, though they go on clinging.*

*Hard to be uncertain, afraid and divided,*

*hard to feel the depths attract and call,*

*yet sit fast and merely tremble -*

*hard to want to stay*

*and want to fall.*

From the poem ‘Yes, of course it hurts’ by Karin Boye. Translated into English by David McDuff (Boye, 1994).

The Swedish poet Karin Boye wrote these lines about a hundred years ago. Since then, they have been widely quoted, usually at school graduations or other life-changing moments for young people, as a metaphor for the transition from childhood to adulthood. However, if we are given the privilege of a life rich in years, it is not only during our youth that we will experience life-changing events. A significant proportion of such events occur during later life due to shrinking social networks (Wrzus et al., 2013), decline of functional capacity (Beard et al., 2016) and an emerging need for support in daily activities (von Berens et al., 2024). In Sweden, turning 65 means the possibility to apply for publicly funded eldercare (Socialstyrelsen, 2023), although many would not define themselves as old even well into this stage of life. Despite generous home care subsidies (Schön & Heap, 2018) and evidence that timely support interventions can slow further decline due to ageing (World Health Organisation, 2017), many Swedes who enter home care after the age of 65 do so only when their needs are already extensive (Meinow et al., 2026). This study explores older adults’ experiences in the phase leading up to the decision of first-time home care.

### Background

Swedish eldercare is a largely tax-funded system that provides services to older adults who needs support in daily life. The most common form is home care, which is provided in ordinary homes by the municipalities (Äldreförvaltningen Stockholms stad, 2025). Individuals pay a fee capped at €235 per month (Socialstyrelsen, 2024), which is determined solely by the individual's income and does not take family resources into account (Silpilä, 2019). Additional reductions are available for those with very low incomes. A municipal need assessor conducts an investigation focusing on why the individual requires assistance and which activities they can and cannot manage independently, to determine the type and extent of home care to which the individual is entitled (Stockholms stad, 2009, 2021). The assessment takes a holistic perspective and includes an evaluation of the individuals’ ability to perform daily activities and their cognitive capacity. The vast majority of applicants are granted some form of support (Municipality, 2025). Services may include support with instrumental activities in daily living (I-ADL: grocery shopping, house cleaning, laundry and meal preparation) and personal activities in daily living (P-ADL: bathing, dressing, toileting, indoor transferring and eating) (Schön & Heap, 2018). From the user's perspective, a distinctive feature of the Swedish eldercare system in an international context is its high level of accessibility, supported by a comparatively simple application process (Peterson & Brodin, 2022) and relatively low care-related costs (Schön & Heap, 2018; Silpilä, 2019). Furthermore, the pathway from application to the provision of home care is generally characterised by a high degree of coordination among the actors involved, including needs assessors, home care providers and, when required, healthcare professionals and clinics (Peterson & Brodin, 2022; Socialstyrelsen, 2012).

Although Swedish eldercare is, by international standards, extensive and accessible to all who require support, public debate frequently highlights a range of challenges that may shape individuals’ perceptions of such services. High‑level political ambitions for high‑quality eldercare, emphasising person-centredness, self‑determination and participation (Peterson & Brodin, 2022; Socialstyrelsen, 2012; SOU 2020:19), alongside post-pandemic calls for sustainable staffing, competence and organisational structures (Wigzell, 2020), create a persistent tension in the face of substantial financial cutbacks to service provision.

Some recent Swedish qualitative studies confirm that people using home care hold negative perceptions. Experiences of losing control over one’s life have been described as occurring when home care staff focus on instrumental solutions for practical tasks, overshadowing the individual’s need for interaction, attention and an individualised approach (Jarling et al., 2018). Older people also describe setting aside their own needs and thus accepting a lower quality of care when they perceive that staff lack the time to meet their wishes in a personalised manner (Westerberg et al., 2017).

Meanwhile, there are also studies that emphasise positive aspects highlighted by older adults using home care, such as the potential of the human encounter with staff for developing positive experiences. Having time for dialogue and time to build relationships with staff is perceived as promoting a sense of independence and control in life, despite functional disabilities (Olsen et al., 2022). The sense of autonomy and self-determination also contributes to the perception of good quality of the home care services received (Olsen et al., 2022).

The link between building positive relations with staff and the possibility for older people to be involved in everyday decisions has also been highlighted as important in other studies (Bölenius et al., 2023; Ottenvall Hammar et al., 2014; Silverglow et al., 2022). Additional research foregrounds the distinctive role of individuals’ contributions to the development of such positive relationships and how these in turn also are associated with positive experiences of services (Harnett et al., 2024; Möllergren et al., 2025; Möllergren, 2026).

In the context of this study, focusing on the phase leading up to first‑time home care, older adults’ preconceptions and related concerns shaping the decision remain poorly understood and this specific scope has received limited attention in international and Swedish research. To our knowledge, the only qualitative studies with a focus of first-time experience of home care were conducted in Sweden (Janlov et al., 2005, 2006). They found that the need assessments largely focused on the practical and physical difficulties of everyday life, while the social, existential, psychological and medical needs were underprioritized and the perspective of significant others largely absent (Janlov et al., 2006). Entering home care for the first time was perceived as a major changeover with existential implications. Family, friends and other close relationships were the preferred option when study participants turned for support. They applied for and entered home care with strong feelings of resistance (Janlov et al., 2005), perceiving a threat to losing the context of belonging and continuity with loved ones (Janlov et al., 2006).

What we define as the phase of life leading up to the decision of first-time home care in this current study is the period from the initial recognition of a decline in functional capacity that results in a need for support—either self-perceived or noted by others—to the individual’s own decision that home care is needed. This is a perspective that is similar to that of Janlöv et al. in the beginning of the 2000s (Janlov et al., 2005) and interesting to re-explore now that more than 25 years have passed.

### Theoretical standpoints and purpose

In this study, we have an overarching critical realist ontological and epistemological starting point (Maxwell, 2011). By this we mean that research and scientific knowledge are inherently dependent on the context in which findings are depicted, as well as on the prevailing understandings of reality that vary over time and across situations. We therefore see this study as a contribution to understanding how the life situation and needs can be experienced when reaching the decision to apply for home care for the first time. This contribution is coloured by our own and our participants' understanding and expectations, which determine the qualities and nuances of the analysis highlighted. The aim was to explore how older people experience the phase of life leading up to the decision of first-time home care. In the following section, we introduce the middle‑range theoretical approach employed to support and interpret our inductively derived analysis.

### Research questions

1. How do older people perceive their life situation and care needs in this context?
2. What are their main considerations and what do these considerations mean for the experience of approaching the decision to apply?

### Conceptual framework

To support our interpretation and present our analysis in a clear and accessible way, we used Meleis’ transition theory in nursing (Meleis, 2010) as a sensitising framework. It stems from role theory and offers a framework to interpret how we as individuals pass through life and transition into and out of different roles. These processes depend on our relationships, expectations and abilities. They give us the opportunity to incorporate new knowledge, change our behaviour and redefine ourselves in relation to our social context when familiar roles are no longer sufficient. Transitions can be conscious and voluntary but also non-reflected or unwanted, depending on the expected rewards and costs of leaving a role or acquiring a new one. Transitions promote well-being and healthy development but can sometimes be challenging, highlighting individuals’ unique vulnerabilities. Transitions start with a marker event or a turning point and are evolving through three different phases: “a period of endings”, “neutral zone” and “new beginnings” (Meleis, 2010). In our study, we define experiencing a transition as the redefinition of a person’s identity as independent, linked to the realisation of a need to accept home care.

## Methodology

This was a qualitative interview study using an experiential and inductive approach to reflexive thematic analysis (Braun & Clarke, 2024).

### Setting

Stockholm County consists of 26 municipalities, of which Stockholm Municipality is the capital of Sweden. The county is populated by 2.5 million inhabitants and almost one sixth are 65 years or older (Statistikmyndigheten SCB, 2024). Twelve percent of the population aged 65 and older use some form of formal eldercare services (Socialstyrelsen, 2026), of which the majority are women and live alone (von Berens et al., 2024). Half of those receiving eldercare also receive support from family or friends on a weekly or daily basis (Meinow et al., 2026).

### Participants and recruitment

Participants were aged 65 or older, had received a first-time home care grant in Stockholm County in 2024, and maximum experience of receiving the services for three months. We recruited the participants with the help of home care provider organisations and pensioners’ associations. One person was recruited through our research network but had no personal relationship with the researchers. As the recruitment process was conducted primarily with the assistance of home care providers and pensioners’ associations across all 26 municipalities, it was not possible to determine the exact number of individuals who were approached. Individuals with severe cognitive impairment or who lacked capacity to independently decide about participation were not contacted. Among the recruited individuals, one man and one woman declined participation. In one case, the individual reported feeling overwhelmed by the number of care-related visits resulting from the health condition underlying the need for home care. In the other case, no reason was given.

We purposely included both men and women and people with varying levels of care needs. Participants with less needs were granted services only for I-ADL and those with more extensive needs were granted services both for I-ADL and P-ADL. In total, 14 individuals residing in six municipalities were included. Eleven out of 14 participants were women and ages ranged from 67 to 94 years. All lived in ordinary housing but one also had access to shared facilities (such as common lounges) in a so-called service apartment complex for older people. All but one had Swedish as their native language. Years of education ranged from 7 to 18 among participants. More than half lived alone, slightly less than half were widowed, and one third were in a relation (co-habiting or not). All participants claimed to receive informal support from family or friends daily and almost half used private out-of-pocket paid cleaning services.

Slightly more than half of participants received home care services for both instrumental activities in daily living (I-ADL) and personal activities in daily living (P-ADL), while the others received assistance with I-ADL only. They had received services between ten days up to three months and the mean number of weeks from debut in home care to interview was six. All participants except two received ongoing services at the time of their interview.

### Ethical considerations

The study was approved by the Swedish Ethical Review Authority (approval number 2023-06089-01) and conducted in accordance with the Helsinki Declaration (World Medical Association, 2024). Participation was voluntary and without compensation. In accordance with the standards established by the Swedish Ethical Review Authority, participants freely provided written informed consent prior to the interviews (*Supplementary material 1 and 2*).

### Preunderstanding, knowledge capital and researcher reflexivity

All researchers are women, live in Stockholm and speak and write Swedish fluently. All but one are born and raised in a Nordic country with similar welfare systems. The first author (PAS) has a background in nursing and homebased primary healthcare. The second author (ÅvB) has a background as a dietitian with experience from geriatric care. The third author (BM) has a background in social sciences and gerontology. The fourth and fifth authors (AL and JA) are public health specialists and social gerontologists.

The idea and design for this study were created by ÅvB, BM and JA as part of a doctoral project aimed at improving the quality of eldercare. PAS led the work of formulating interview questions, conducted the interviews, outlined the analysis in collaboration with the co-authors, and provided updated versions of the manuscript throughout the reporting process.

After many years as a district nurse in home healthcare and primary care, PAS has both theoretical knowledge acquired through education and “practice wisdom” (Ide & Beddoe, 2024) about older people with complex care needs and home care use. This enabled a narrative for this study with an explicit nursing perspective on aging and support needs. This perspective was further shaped by the theoretical and clinical backgrounds of the co-authors, for example, by the interest in how eldercare support services can be best tailored and made optimally accessible. This interest origins from several years of previous research and investigations of the municipal and regional care systems in Stockholm and in clinical experiences of caring for older people when they, sometimes reluctantly, enter home care. As authors, we have also invested in this work, our accumulated experiences of Swedish home care throughout our lives - as daughters, friends, and grandchildren of older people using these services. We acknowledge that other experiences and different forms of knowledge could have generated alternative interpretations and brought additional valuable insights to light.

### Dataset generation

We developed a semi-structured interview guide and pre-tested it for accuracy and relevance together with two older adults with experience of home care use (Supplementary material 3). Three questions correspond to the purpose of this study (Table I).

**Table I.** Interview guide.

Semi-structured questions
What does a typical day look like for you?
What was your living situation and health like before applying for home care?
What considerations did you make before applying for home care?

The first author, PAS, conducted the interviews in an open, conversational manner and, at the participants' request, in their own homes. She took field notes in close connection to each interview to document context characteristics to facilitate the interpretation process.

PAS interviewed all participants once, and each interview lasted between 44 and 107 minutes. Three participants were accompanied by a relative. These relatives sometimes provided supporting information, such as chronological details, with the consent of the participants. PAS audio-recorded the interviews and contracted a professional service to transcribe them verbatim. PAS checked the accuracy and quality of the transcription and when necessary, she corrected texts in accordance with the audio files. At the start of the coding process, she pseudonymized the transcribed interviews, removed identifying information, such as personal names and geographical terms and uploaded them into NVivo software.

PAS coded the text progressively in three rounds in parallel with the recruitment and interviewing process. When 14 interviews were coded and systematised for a first overview, we assessed information power collectively. While efforts to increase male participation yielded limited results, we considered the dataset rich, offering substantial empirical depth to support a comprehensive analysis of older adults’ experiences in accordance with the study’s purpose.

### Analytic method

We used reflexive thematic analysis (RTA) as a method and philosophical approach for our processing of the data (Braun & Clarke, 2019, 2024). RTA provides a procedural framework where the synthesis of meaning is central (Byrne, 2022). This is well aligned with our theoretical approach to people’s experiences of their home care debut as grounded in personal views as well as prevailing sociocultural norms. RTA has offered us support to create a rich, detailed, and nuanced qualitative analysis of the experiences of applying for home care for the first time, and to continuously and reflexively process data in relation to theory, literature and our own knowledge and understanding (Braun & Clarke, 2019; Braun et al., 2022). We used an experiential orientation throughout the research process (Braun & Clarke, 2022) and an inductive approach for the coding and development of themes. We meant to be sensitive to both semantic and latent aspects of meaning in the data. While the study was primarily guided by the RTA guidelines (Braun & Clarke, 2024) to ensure quality and rigour in the analytical and reporting process, we also applied the COREQ checklist (Tong et al., 2007).

### The analytic process

We familiarised ourselves with the data by listening through and discussing the interview content. When less than half of the interviews were conducted and transcribed, we drafteda coding of five interviews and jointly discussed possible, preliminary patterns. We consolidated our focus on content and aim for this study and clarified the delineation of the data set aside for other, forthcoming studies. We identified codes and themes within two broad areas; a) how life had changed, and b) the process of accepting the need for home care. We worked in repeated interpretation cycles where first author PAS developed interpretive draftsin the form of codes, themes, and text, which were then presented to the research group and jointly discussed. By alternately reflecting on the more semantic and the more latent levels in the dataset, we restructured the text continuously until the descriptions of how the different parts of the phenomenon relate to the whole were consistent and clarified their mutual relationships. We conducted reflexive journaling throughout the process. In writing the article, we used the free version of DeepL Web Translator and Microsoft Copilot, large language model (LLM) tools, to refine the text in English. However, we have produced all of our creative content ourselves.

## Results

**Figure 1.**
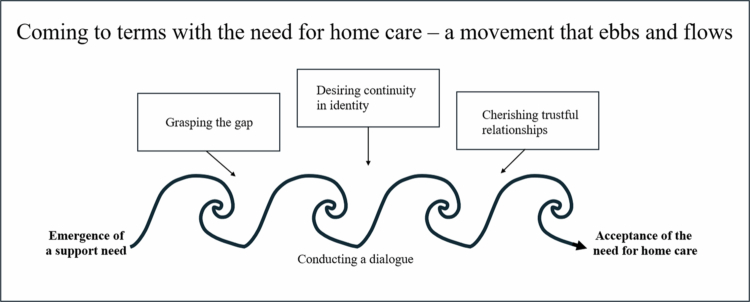
Illustration of the relationships between theme and sub-themes.

**Figure 2.**
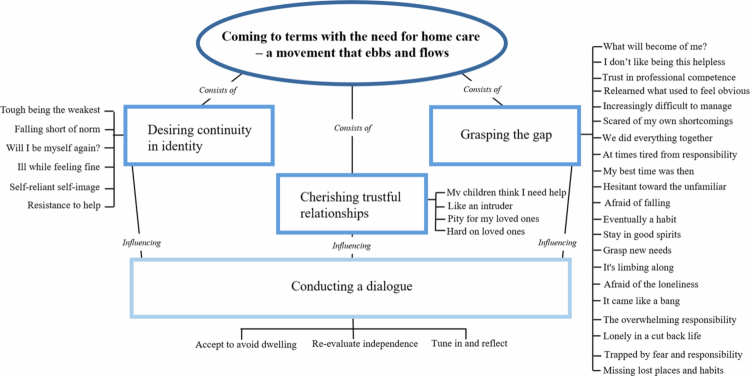
Concept map showing how theme, subthemes, and codes are organised hierarchically.

We aimed to explore how older people experience the phase of life leading up to the decision of first-time home care. The overall theme *‘Coming to terms with the need for home care—a movement that ebbs and flows’* seeks to capture the meaning and nature of how participants interpreted, reinterpreted, identified and defined their needs for home care: from a hunch that they might need it, self-perceived or noted by others, to the conviction that the personal situation had changed in such a way that home care was inevitable. Participants described this as a process, where different but not necessarily opposing forces influenced and mediated how their view of the need for home care services developed. Participants reported how they gained a growing acceptance of their need according to a non-linear but fluctuating movement, reminiscent of the ebb and flow of water. Their experiences arose from reflection on changed external circumstances, which we describe in the sub-section ‘Grasping the gap’. It was also influenced by internal and external triggering factors, which we outline in the sub-sections ‘Desiring continuity in identity’ and ‘Cherishing trustful relationships’. Finally, in the sub-section ‘Conducting a dialogue’, we elaborate on the dynamic nature of this experience. The overall theme is based on 35 codes (Supplementary material 4). An overview of theme and sub-themes is presented in Table II and the relationship between them is illustrated in Figure 1. Figure 2 shows how the study's main theme, sub-themes, and codes are organised in a hierarchical structure.

**Table II.** Theme and sub-theme summary.

*Theme Coming to terms with the need for home care—a movement that ebbs and flows.*	
Sub-theme	Characteristics
Grasping the gap	How changes in life circumstances create a foundation for new insights into the needs for support and for home care
Desiring continuity in identity	How previous social roles may influence the emotional understanding of new needs and the ability to come to terms with the need for home care
Cherishing trustful relationships	How advice from trusted individuals, along with efforts to maintain good relationships with those who matter most, may trigger and drive the process of understanding and accepting the need for home care
Conducting a dialogue	How the process of coming to terms with the need for home care fluctuates according to a non-linear movement of an internal and sometimes external dialogue

### Grasping the gap

A common feature described by the participants was a pervasive sense of limitation — social, mental or psychological — in which everyday habits and activities became increasingly constrained. Over time, participantscame to perceive their previously independent roles and identities as being altered or diminished. These changes in life circumstances could unfold gradually or occur abruptly following a single event.

With declining functions, participants described a shrinking life space with behavioural changes resulting from giving up previous habits, social relationships and places to visit. The opportunity for privacy, recreation, social interaction, and spontaneity in daily life deteriorated. Grasping the gap between former abilities, new support needs, and the resulting need for home care, was a gradual process. Although not all participants described the decision to apply for home care as life-changing, the need for formal care still evoked a sense of discomfort.

*Well, there really weren’t any considerations to make. It was completely obvious that I wouldn’t be able to manage on my own. And then it’s strange, really. Because instead of doing everything myself, it’s someone else who has to help you with it, right? So it becomes an adjustment in that sense… But it was so obvious that I needed it, so it wasn’t that difficult after all. The only thing was […] the personal hygiene, you know. Having someone wipe your bottom after you’ve been to the toilet and so on. That was… That’s not exactly something you take lightly, but…* (Helena, 73 years)

By some participants, however, this process of coming to a decision lacked a given direction and although burdened by the stresses of care needs and limitations, home care was not their obvious or immediate choice. Some participants, themselves, struggled to understand their support needs. Some participants also struggled to get the people around them to understand what their functional impairments meant to them in their daily lives.

*It's like being a child again, putting on a pair of shoes for the first time. […] And it's not about tying them but just putting them on. […] You don't talk about it very often because it's quite difficult for people to understand. And difficult to understand yourself, too, actually. Because you don't think about the worst times, you think about the best times, perhaps.* (Anders, 67 years)

To other participants, grasping the gap and gaining insight into their need for home care was a process not primarily complicated by reaching intellectual awareness about the impaired ability to manage as before. Instead, it involved psychological or emotional steps on the path from realising new support needs to accepting a need for home care. Margareta provided an example of being aware, at some level, of her need for support, while at the same time struggling with thoughts that conflicted with this:

*I suppose I’m stubborn enough to want to manage on my own. But [the neighbours] all say down there, ‘We can see that you’re not managing much on your own anymore. You’re not functioning. You can’t hear what people are saying. You can’t go shopping anymore. You can’t use…’ They tell me what I can’t manage. But in my mind, I can manage it — except… I can’t. I have to admit that to them in the end.* (Margareta, 82 years)

### Desiring continuity in identity

Some participants described how they had built an identity as independent throughout their lives. This was sometimes presented in positive terms as an ideal to which participants were loyal, something to which they were trained to aspire. Some wished to maintain an image of inner strength and persistence. Others were devoted to a lifestyle as courageous and adventurous and wished to live by the motto that people in general have an inherent capacity that is greater than they think, if only they have the courage and energy to take on the challenge.

*I believe that everyone is capable of more than they think. I’m completely convinced that I’m right about that. And of course, that applies to me as well. So I try.* (Anders, 67 years)

Participants who themselves had worked in health and social care all their lives described themselves as strong people, even if they were struggling with some illness or disability. It was important to them that they had always managed on their own and throughout their lives been needed by others; their children, their ageing parents, their sick partners and all the people they had taken care of professionally. They perceived themselves as helpers. This was a virtue and an act of solidarity, a social contract with society and fellow human beings. Maintaining autonomy and the virtue of helping others could therefore be factors strongly associated with an image of a life without home care services.

*That's what my mother taught me and my siblings. That we should manage on our own. We shouldn't have to ask for help. We should be helping others.* (Margareta, 82 years)

Although obviously ill, in terms of medical diagnoses, some participants were keen to point out aspects that contributed to well-being and emphasised health factors that were particularly important to them, despite diagnoses. They discussed issues related to the concept of health and the values they considered important for well-being.

*At the same time, I am actually very happy today, even though it has of course been difficult. But you discover that, especially since I got Parkinson's, I've made friends that I wouldn't have made otherwise, who are close friends. […]. Although I have my own car, [a friend] picks me up and drops me off at table tennis, for example, twice a week. […] And he always looks so happy. And I ask, “Why do you look so happy?” “Well, because I get to see you.” So, you understand? I had never heard such nice words before, before Parkinson's that is.* (Anders, 67 years)

For some participants in our study, the gap between before and after the loss of functional ability threatened a loss of the very essence of who they were. They feared being permanently unable to feel and relate to the world in a way that was consistent with the previous self-image as independent. These feelings were not primarily a reaction to the need for home care itself but rather linked to the experience of losing independence. However, as resulting in hopelessness in the form of bottomless powerlessness, desperation and existential anxiety, these feelings had a significant impact on the process of accepting home care.

*I think it's the feeling that I'm just going to disappear somehow. That I'm going to die, that I'm going to pass away or something like that. And I've never had that before. That I feel so helpless. So, I feel, aban... Not abandoned, what can I say...? It feels like I'll never be myself again. The way I was before.* (Gunilla, 71 years)

### Cherishing trustful relationships

Because participants were often very concerned about their loved ones and attentive to whether they felt burdened by providing support, applying for home care could become a way of protecting one’s immediate sphere of meaningful relationships. Some participants described being triggered into an ambivalent process towards accepting their needs because the opinions of their family and friends conflicted with their own. The acceptance of home care could be one of the compromises made together with the person with whom one shared one's life and home. Sometimes it was described as a way of avoiding conflict or disappointments. However, it could also result from the desire to show gratitude to family and loved ones.

*Then I want to show my gratitude to them for thinking of Mom. That Mom should have the best. When they kind of... They've actually been nagging me to apply for home care. So I think it feels a bit awkward to be ungrateful for their consideration.* (Elsa, 88 years)

Another example of how powerful informal bonds between older adults and their significant others can influence the acceptance of first‑time home care was the desire to preserve and protect certain qualities within these close relationships, for instance those related to sexuality. Even participants who were emotionally overwhelmed by the loss of independence and therefore initially hesitant to accept formal home care described feeling relieved by the possibility to receiving help with very intimate daily matters—such as toileting and personal hygiene. These tasks caused them the greatest distress, anxiety, and ambivalence, and although it felt awkward to receive help from strangers, it was still preferred over asking their partner.

*I think about our sex life; how will he see me when all this is over? I mean, we're supposed to resume our life as husband and wife. Should he then think about the fact that he is cleaning... No, I don't want that, absolutely not... No, he just can't do that.* (Gunilla, 71 years)

Other participants described how feelings of shame and guilt towards family and friends could drive the wavering back and forth in the process of accepting the need for home care. They did not want to be a nuisance and burden in important social relationships, and tasks that they could not handle in their own daily lives, they did not want to affect family and friends in *their* daily lives.

*Damn, it's hard. Once they've left, I feel ashamed of having asked them for help. […] They're not young themselves. They have their own lives and families and stuff like that. I feel like an intruder.* (Margareta, 82 years)

In contrast to those participants whose view of applying for home care conflicted with that of significant others, some participants perceived the input from others as empowering and as contributing to feeling better informed in their decisions. These participants were keen to understand how people who were important to them in their lives perceived their new situation and asked for the opinions of family, friends or professionals of trust.

*I couldn’t do anything else [than apply for home care]. Because that’s when I realised that I can’t… it just isn’t possible. I can’t manage on my own. […] And I can’t be sure that my partner could help me either. I said, ‘How will this work?’ [The need assessor answered,] ‘Well, it will probably be fine, because you’ll get help at home and you’ll get help several times a day.’ And it really was many times a day that I was told I could receive help.* (Barbro, 70 years)

### Conducting a dialogue

Grasping the gap between previous independence and the actual need for support and home care was often described as a dialogue. In such dialogue, which participants conducted internally but sometimes also could involve others, some participants tried to calm their annoyance at no longer being able to perform previously natural everyday actions and remind themselves to stop dwelling and move on.

*Then I can get angry at myself for not being able to [water the flowers]. […] Because I managed it myself before. But at the same time, I realise up here... “Yes, but you're not doing it.” […] Then I think, “Damn, now I can't reach them.” But then I go, “Yeah, but face it, you can't reach them.”* (Bodil, 78 years)

Some participants described how they, at the beginning of a rapid deterioration in their health or functional capacity, not only engaged in an internal dialogue, but also acted out their feelings, in affection. They described how they made hasty decisions and involved family and friends in an attempt to return to the daily life they were used to, a life that was no longer possible. Gunilla’s example at hospital discharge, following treatment for severe fall-related injuries, illustrates how she attempted to escape her overwhelming feelings through denial and enacted her ambivalence in an external dialogue with her sister and hospital staff. In the following quote, she recounts the situation at the hospital, in which she declined any form of further support:

*The first thing I remember is that I didn’t want any help, and that I was going to move in with my sister. [….] I was worried about stairs and things like that. Then my sister said ‘You can come and stay with me.’ Since they live in a house [without stairs]. She said ‘I’ll take care of you.’ […] And then I just said [to the hospital staff] ‘No, I’m going home, or to my sister’s.’ It was like… I was completely blocked. I’ve never experienced anything like this before.* (Gunilla, 71 years)

Participants adapted to the necessity of receiving home care in their respective emerging situations, with varying degrees of pragmatism and differing needs for reflection, dialogue, and time to come to terms with it. Not all seemed convinced that home care was the appropriate solution to their needs at the time that they decided to apply. For several, the process required extended periods of emotional or psychological work to process its implications. Some had agreed to apply for services primarily as a compromise with relatives or friends, while still reporting that they were engaged in reflections about their sense of self in relation to their emerging support needs.

*I rely a lot on myself and I’m not very good at… well, I think I’ve been bad at accepting help from others. I do think that. So that’s what I mean when I say that this period ahead of me will show a lot — how well I can manage that. Accepting help from others.* (Anders, 67 years)

## Discussion

The analysis in this study gives insight into how older people may experience the phase of life leading up to their decision to apply for home care for the first time; how they come to terms with this new need in life. The pervasive nature of this experience lies in the pendulum-like movement of acceptance when efforts are made to align emotional will to remain independent with intellectual understanding of changing support needs. Altered life circumstances may contrast to established social roles and lead to more difficulties for some than for others to adapt while maintaining a strong and distinct sense of identity. A desire to cherish relationships with those who matter most may trigger and drive this process, in both internal and, at times, external dialogue. We subsequently further develop and discuss our inductively derived analysis supported by transition theory in nursing (Meleis, 2010).

Participants who were more emotionally engaged by applying for home care were torn between how they wanted their situation to be, how they remembered it once being, how they were used to thinking about it, how they thought others perceived it, how they had been brought up to understand it, their fears about how it might develop, and the understanding they gradually developed when confronted with it in everyday life. In transition theory (Meleis, 2010), this pattern of weighing benefits against drawbacks and mentally oscillating as participants moved toward acceptance can be understood as the individual being influenced by different push-and-pull forces. An uncomfortable friction arises as the individual continues to deal with life using the same attitude, resources and strategies that the original role provided. Drastically new circumstances that are provoked either gradually or all at once thereby force the individual to struggle for restored balance in life by creating a new way forward. The instability becomes the very runway of departure for the individual’s transition and provides fertile ground for new knowledge, new behaviours and, in some cases, a redefinition of identity (Meleis, 2010).

In this study we show how a self-image as strong, healthy, caring for others, stubborn and adventurous was difficult to reconcile with assuming the role of someone in need of help. Participants underwent this process of change reluctantly, consistent with Meleis (2010), who note that transitions can sometimes be involuntary. Although participants might have had an intellectual understanding of the nature of their emerging needs, they often remained emotionally attached to their previous roles and the ideal of independence. This attachment could lead to a perceived capacity to manage everyday activities that did not align with their actual abilities. According to Meleis (2010), push-and-pull forces that underpin involuntary role exits are often shaped by stigmatising elements. The use of home care can hardly be considered as stigmatised among older adults in Sweden—after all, there are no alternative eldercare systems besides the public one in Sweden and these services are used by a vast majority at some point during later life (Meinow et al., 2020). However, its association with declining functional capacity, aging, and dependence on others may nonetheless complicate acceptance of home care for individuals who view independence as a positive ideal in contrast to these processes.

The struggle to maintain continuity in identity in the context of first-time home care use can be interpreted through theories of social behaviour. What is perceived as familiar is linked to identity and self-image, which means that sticking to familiar contexts and activities may create balance in later life when older adults are faced with disruptive events (Atchley, 1989). Alternatively, according to the theory of selection, optimisation and compensation (SOC) (Ikner et al., 2026), some participants’ strong desire to manage without home care, or as little home care services as possible, can be interpreted as an act of selection or compensation made in order to maintain a self-image of being strong, stubborn, healthy, or adventurous. One example of how this often comes at the expense of a certain level of comfort or involving a degree of risk-taking in everyday life is how Anders, 67 years, in an earlier quotation in section ‘Grasping the gap’ describes how he, despite sometimes significant difficulties, continued to put on his shoes by himself before evening walks.

Janssen et al. (2012) argue that the experience of being in control of one's own life is crucial for people to be resilient as they age. This means that identity needs to develop in such a way that the need for help is incorporated as something positive and empowering. Maintaining a sense of independence does thus not have to be at odds with the need for home care. Accepting the need for support can instead be seen as a sign of an ability to embrace new strategies for coping with changing and deteriorating conditions during aging.

The pursuit of maintaining one's lifestyle and sticking to strategies for managing one's daily life independently despite age-related functional impairment can also be an important motivating factor in creating meaning in life in old age. This has been shown by Machielse (2025) in a study on socially isolated older adults’ experiences. Pride in being resilient and coping despite difficulties may help boost one’s self-image by adding positive values and creating a coherent life story when the lack of something to look forward to in life is perceived as significant.

Several participants in our study described how they exhausted all personal resources and sometimes enlisted the help of family and friends to find new, innovative ways to compensate for their needs, before arriving at the conclusion of applying for home care. This is reminiscent of previous research on the experience of deciding on first-time home care, where Janlov et al. (2005) even described the struggle to avoid dependency and home care as an expression of existential concerns. Losing abilities, strategies, the feeling of control in life and a part of what characterised the personality on a deeper level, relates to the concept of existential distress or demoralisation, often discussed in palliative care. Existential distress is the psychological friction that arises and creates emotional pain when certain circumstances in life challenge us in an overwhelming way (Grech & Marks, 2016; Philipp et al., 2025).

Our analysis highlights the considerable influence that the views and input of significant others may have in the process of older adults coming to terms with a need for home care. Research shows that cherishing contact with loved ones becomes particularly important as we get older and can thus contrast with other things in life that instead become less important or difficult to achieve with age. Gradually diminishing opportunities to be mobile and capable, to maintain commitments and long-term plans, and the loss of social status in society are examples of what can challenge the experience of meaningfulness. However, close relationships with family and friends often remain highly valued. Maintaining contact with younger generations of relatives has for example been described as contributing to a sense that one’s own existence during aging is important in a larger context and serves as a link between previous and new generations (Jonsén et al., 2015; van Rhyn et al., 2022).

From a role transition perspective, the individual’s social circle is also acknowledged as an important potential influencer, because of its assigned value. The very definition of the dynamics of role change, as formulated by Meleis (2010), includes the interdependence between the roles of ego and the roles of significant others. In a social circle, the role of one individual can relate to the role of another individual as being its counterrole. This means, the behaviours and identities that ego manifests in the social interplay with others will fulfil expectations and needs of the counterroles of others with whom ego has relational bonds. Therefore, people often go through transitions in response to the transitions manifested by others.

The pattern of dynamic change within a given social context is relevant to better understanding the experiences of older people when approaching acceptance of home care. In particular, it suggests that significant others have the potential to have both a stabilising and triggering effect on older adults’ change processes. The participants in this study had different perspectives on the meaning of significant others in the context of first-time home care. Some bowed to the explicit or implicit demands of family and friends to conform to their views and although not necessarily agreeing at once, they simultaneously received the spark of a thought or new idea that home care was needed from this interaction. Others were affected by feelings of shame linked to the help given by significant others or were influenced in their home care decision by a strong desire to protect particularly intimate features of a close relationship. Still others were actively seeking out the opinions of others and appreciated them as helpful advice that strengthened them in the process of coming to a decision. Even though these experiences were thus shifting in nuance, a common trait shared across them was the great impact that trustful relationships had as a driving force. Both in the process of accepting new support needs and ‒ linked to this ‒ of accepting the need for home care.

The fluctuating, non-linear movement we observe when analysing the experience of coming to terms with this need is linked to the individual’s internal, and sometimes external, dialogue. Ueland et al. (2018) have also argued that personal change develops as a movement accompanied by an internal dialogue within the individual. By referring to the theoretical contributions of Sören Kierkegaard, (1996) and Katie Eriksson (Näsman, 2020), the authors define love and suffering as the forces from which human change processes originate and highlight the importance of longing as a driving force for creating experiences of health. In the event of a life change that has a profound impact on feelings and thoughts about existence, the internal dialogue serves to clarify and create transparency about the process; the individual’s motives and hesitations and becomes a path to reconciliation. It is an opportunity for individuals to face their own vulnerability, be honest, and develop knowledge about who they are (Ueland et al., 2018).

The changing of social roles is a process that can run over prolonged periods, depending on personal vulnerabilities and external circumstances. Transitions can lead to positive outcomes for the individual, such as new skills and behaviours that facilitate managing the newly acquired role. However, they can also be inhibited or interfered with, and thus become extended or remain uncompleted (Meleis, 2010). Anders, 67 years, illustrates this at the conclusion of the results section by articulating his reflections on how his decision to apply for home care may ultimately influence his acceptance of receiving such support in practice. Our synthesis suggests that people who experience strong emotional or psychological reservations about accepting support through home care may not have completed this transition when they decide to apply for services. From a transition theory perspective, some older adults may thus find themselves in an uncompleted transition, initiating home care services while remaining deeply ambivalent about the decision.

### Strengths and limitations

A key strength of this study is our focus on older adults’ first-time experience of deciding on applying for home care; an exploration of the early phase of the process of becoming a care recipient that is underexplored. We aimed to capture the experience as close to the introduction of services as possible, which is why we interviewed participants with experience of first-time use between tendays and three months. We found that allowing up to three months of experience was a practically feasible compromise, as deciding on and receiving home care for the first time often happens in a turbulent time in life, sometimes involving energy-intensive phenomena such as pain, worries and interactions with several healthcare providers. Moreover, a certain time distance to what can be experienced as an undesired or even disruptive change can entail the opportunity to reach more reflective conclusions. However, this time span may be considered long given our ambition to capture older people’s recollections of how they reasoned prior to their decision. We acknowledge as a limitation of this study that we were unable to capture the participants’ thoughts preceding the decision to apply while they were still fresh in mind, and before they may have been influenced by the experience of receiving home care services. The retrospectively narrated experiences, may, for example, have resulted in fewer participant accounts of apprehensions about home care use. Concerns portrayed in the media, discussed by the general public, or linked to specific individual conditions, are issues that participants may have expected to significantly influence their first-time use but that they may have forgotten if their actual experiences then turned out to be different.

The authors’ professional backgrounds and extensive experience of Swedish eldercare likely shaped the interview focus and analytic interpretations. While this facilitated contextual understanding, it may also have foregrounded care- and nursing-oriented readings of the data. We sought to counterbalance this through ongoing team discussions and active consideration of alternative interpretations.

A limitation is that we were unable to capture the opinions of the most vulnerable individuals and those with severe cognitive impairment. We have, however, in other respects managed to generate data rich in variation, involving experiences of varied socio-economic backgrounds and educational levels as well as of different levels of support needs.

From a gender perspective, it is a limitation that only three men participated in this study. Women’s and men’s experiences of performing household chores and personal care are known to differ at the population level, and it is possible that their experiences of needing support with these types of tasks and their process of coming to terms with the need for home care also differ. In addition, as the research team is entirely female, some gendered perspectives may have been less visible in the analysis. Men’s experiences of first-time home care remain an important area for future research.

### Final reflections

Our analysis suggests that the individual’s decision to apply for home care for the first time arises from a fluctuating internal process experienced with reluctance. Navigating this period of ambivalence requires engagement in internal and sometimes even external dialogues as the individual moves from acknowledging emerging needs for support to eventually coming to terms with the necessity of home care. At the same time, the individual is influenced by various push-and-pull forces rooted in values, norms, and the intricate social interactions that build trustful relationships. Our analysis further shows how the process may involve a desire to maintain continuity in one's identity by upholding values beyond everyday independence. This synthesis also suggests that first‑time home care decisions may emerge as a compromise with significant others, thereby reinforcing existing strong relational bonds. For some individuals, coming to terms with the need for home care entails substantial mental and emotional reorientation, and, subsequently, a transition into new or changed social roles. We found that individuals who, due to strong emotional and psychological reservations linked to the need of accepting support through home care, go through extensive identity redefinition may not necessarily have completed this transition when deciding to apply. This suggests that some older people may start using home care services when still profoundly ambivalent in this decision.

Our study provides insight into why some older adults, although eligible for support programmes and in need of assistance, do not necessarily seek out available resources. This adds a new dimension to existing theories on older people's help-seeking behaviour (Wacker & Roberto, 2016; Wulfse-Huisman et al., 2025). Highlighting the interplay between psychological and sociological mechanisms, our analysis demonstrates that what may seem paradoxical from an outside perspective can be experienced as meaningful and logical by older adults in this Swedish context as they navigate their support needs and make a decision to initiate home care. Moreover, classic (Lawton & Simon, 1968) as well as more contemporary theory (Menassa et al., 2023; Wahl et al., 2012) on how social structures, values, and individual experiences interact underscore the relevance of this interplay for shaping, and eventually, transforming, identity and social roles in later life, as reflected in the patterns identified in this study.

#### Added value of a transition Theory–guided discussion of first-time home care

Our interpretation of older adults’ experiences of reluctantly moving towards acceptance of home care highlights how transition theory in nursing can guide to new knowledge. In the process of aging, individual, psychological processes of change are closely linked to functional ability and performance of activities of daily living. These changes sometimes occur under drastic circumstances. However, they are also continuously, and inevitably underway as physical and cognitive conditions can change little by little. We suggest that transition theory in nursing offers a valuable framework to distinguish and thus facilitate understanding of different stages involving such processes related to eldercare use, and the emotions, identities, values, intellectual processes, strategies, and behaviours concerned. Although Meleis’ theoretical framework (Meleis, 2010) has previously been applied in studies addressing topics closely related to home care use (Coffey, 2012; Gama et al., 2025), it has, to our knowledge, not yet been used to support reflections on the process of approaching a decision regarding first-time home care. In that sense, this theoretical lens for first-time use has provided new insights into a possible interpretation of this significant shift in life.

#### Clinical implications and value for the wider community

The reflections and syntheses of this paper can inform stakeholders at all levels of the eldercare system; policy, management, needs assessment and home care provision. It highlights the gradual process individuals undergo prior to applying, showing that it varies in duration and emotional intensity depending on the individuals’ personal circumstances. This can help healthcare and social services providers, who often come in contact with people who have not yet started home care, in their proactive assignment to facilitate and guide to appropriate support. Considering the possibility that individuals who are particularly reluctant to accept home care despite an obvious need for support may be strongly devoted to their self-image as independent or as a helper can be useful, for example when continued care at home is planned for older adults after hospitalisation. Being aware of the important potential that significant others have as a motivating force is useful knowledge when informing, motivating, or offering home care services to potential first-time users. Ultimately, this study provides insight into older people’s perspectives as they navigate decisions about receiving home care for the first time. It also underscores the importance of staff remaining attuned to the preferences and understanding of the target group through dialogue both before and after home care begins to ensure that formal support is accessible, effective, and appropriately adapted.

In a broader sense, this study can contribute to a more nuanced public understanding of the specific challenges that older people face as their need for home care emerges. It may help older adults who, for different reasons, feel strong resistance to applying even when support needs are extensive. It may also help people, in their roles as family members and friends of older adults, become better prepared to offer support and to understand their own roles in this phase of life.

Although this study was conducted in Sweden, the challenge of tailoring support to individual needs and ensuring that support services are genuinely accessible from the users’ perspective is universal. The findings contribute to our understanding of the complex ways in which individuals approach a need for support and suggest that a desire to maintain independence may play a significant role in shaping the process of acceptance of the need for home care. This challenges the assumption that, in times of limited resources, rigid control systems and centralised definitions of eligibility are effective means of ensuring access to support. In this way, the study contributes to the broader international debate on how eldercare systems can be prepared to meet growing demands in the future.

The strong influence of close relationships in participants’ decisions to accept formal support, despite their initial reluctance, underscores the important role that family and friends can play in the uptake of care services. Even in societies characterised by a strong welfare state and a longstanding tradition of public provision, first-time home care may be far from an obvious choice. These findings highlight the importance of significant others in older adults’ decisions about seeking and accepting eldercare. They also reveal the emotionally vulnerable position of informal caregivers supporting older adults with functional decline, emphasising the need to recognise and address the needs of this group. This is relevant both in Sweden and in countries where families play an even more central role in providing support in later life. It is particularly pertinent in light of the growing reliance on informal care, both in Sweden (Kirvalidze et al., 2025; Rostgaard & Szebehely, 2012) and internationally (Cattaneo et al., 2025; Rocard & Llena-Nozal, 2022).

#### Implications for future research

To provide continuity to our study, we suggest that future research specifically explores whether and how sex and gender influence the experience and how individuals suffering severe frailty or cognitive difficulties perceive the home care debut. In addition, further studies may be needed to understand how the life situation of those with a weak social network influence their experiences of arising support needs and how they reflect upon the need for home care in the absence of input from significant others.

## Supplementary Material

Revised_Supplementary_material_2.docx Revised_Supplementary_material_2.docx

Revised_Supplementary_material_3.docx Revised_Supplementary_material_3.docx

Revised_Supplementary_material_4.docx Revised_Supplementary_material_4.docx

Revised_Supplementary_material_1_.docx Revised_Supplementary_material_1_.docx

## Data Availability

The qualitative interview data are not publicly available and cannot be shared upon request due to ethical and legal restrictions under Swedish law and the conditions of the ethical approval. Sharing the underlying transcripts could compromise participant confidentiality. Pseudonymized quotations supporting the analysis are included in the article.

## References

[cit0001] Äldreförvaltningen Stockholms stad . (2025). *Äldreomsorgens årsrapport 2024* [Annual report on eldercare 2024]. https://meetingspublic.stockholm.se/welcome-sv/namnder-styrelser/aldrenamnden/mote-2025-03-25/agenda/bilaga-1-aldreomsorgens-arsrapport-2024pdf?downloadMode=open.

[cit0002] Atchley, R. C. (1989). A continuity theory of normal aging. Gerontologist (Washington, DC), *29* (2), 183–190. 10.1093/geront/29.2.183 2519525

[cit0003] Beard, J. R. , Officer, A. , de Carvalho, I. A. , Sadana, R. , Pot, A. M. , Michel, J. P. , Lloyd-Sherlock, P. , Epping-Jordan, J. E. , Peeters, G. M. E. E. ( , Mahanani, W. R. , Thiyagarajan, J. A. , & Chatterji, S. (2016). The world report on ageing and health: A policy framework for healthy ageing. Lancet, *387* (10033), 2145–2154. 10.1016/S0140-6736(15)00516-4 26520231 PMC4848186

[cit0004] von Berens, Å. , Wallcook, S. , & Meinow, B. (2024). SNAC Stockholm 2022. En beskrivning av äldreomsorgstagarna och omsorgens omfattning I stockholms stad 2022 [A description of the eldercare recipients and the scope of social care in the city of Stockholm in 2022], *Stockholm: Stiftelsen Stockholms läns Äldrecentrum* . Stockholm Gerontology Research Center.

[cit0005] Bölenius, K. , Lämås, K. , & Edvardsson, D. (2023). Older adults’ experiences of self-determination when needing homecare services—an interview study. *BMC Geriatrics* , *23* (1), 824. 10.1186/s12877-023-04533-6 38066429 PMC10709827

[cit0006] Boye, K. (1994). *Complete Poems* . Bloodaxe Books Ltd.

[cit0007] Braun, V. , & Clarke, V. (2019). Reflecting on reflexive thematic analysis. *Qualitative Research in Sport, Exercise and Health* , *11* (4), 589–597. 10.1080/2159676X.2019.1628806

[cit0008] Braun, V. , & Clarke, V. (2022). Thematic analysis - a practical guide, London: SAGE Publications Ltd.

[cit0009] Braun, V. , & Clarke, V. (2024). Supporting best practice in reflexive thematic analysis reporting. *in Palliative Medicine: A review of published research and introduction to the Reflexive Thematic Analysis Reporting Guidelines (RTARG). Palliative Medicine* , *38* (6), 608–616. 10.1177/02692163241234800 PMC11157981 38469804

[cit0010] Braun, V. , Clarke, V. , & Hayfield, N. (2022). A starting point for your journey, not a map’: nikki hayfield in conversation with virginia braun and Victoria clarke about thematic analysis. *Qualitative Research in Psychology* , *19* (2), 424–445. 10.1080/14780887.2019.1670765

[cit0011] Byrne, D. (2022). A worked example of braun and clarke’s approach to reflexive thematic analysis. *Quality & Quantity* , *56* (3), 1391–1412. 10.1007/s11135-021-01182-y

[cit0012] Cattaneo, A. , Vitali, A. , Regazzoni, D. , & Rizzi, C. (2025). The burden of informal family caregiving in Europe, 2000–2050: a microsimulation modelling study. *The Lancet Regional Health - Europe* , *53* . 10.1016/j.lanepe.2025.101295 PMC12008708 40255934

[cit0013] Coffey, A. (2012). Theory of transition and readiness of older people for discharge from hospital to home, *Paper presented at the 23rd International Nursing Research Conference* . Sigma Theta Tau International. Brisbane, Australia. editor Meleis’.

[cit0014] Gama, L. M. P. , Ribeiro, K. R. C. , Oliveira, J. L. C. , Costa, M. A. R. , Acosta, A. M. , & Souza, V. S. (2025). Transition from hospital to home care: A mixed methods study in light of Meleis's theory. Revista brasileira de enfermagem, *78* (1). e20230357. 10.1590/0034-7167-2023-0357pt 40105528 PMC11913045

[cit0015] Grech, A. , & Marks, A. (2016). Existential suffering part 1: definition and diagnosis #319. *Journal of Palliative Medicine* , *20* (1), 93–94. 27918681 10.1089/jpm.2016.0422

[cit0016] Harnett, T. , Möllergren, G. , & Jönson, H. (2024). The use of home care as relational work: outlines for a research programme. International Journal of Qualitative Studies on Health and Well-Being, *19* (1), 2371538. 10.1080/17482631.2024.2371538 38913083 PMC11198145

[cit0017] Ide, Y. , & Beddoe, L. (2024). Challenging perspectives: reflexivity as a critical approach to qualitative social work research. *Qualitative Social Work* , *23* (4), 725–740. 10.1177/14733250231173522

[cit0018] Ikner, B. , Baltes, B. B. , & Rudolph, C. W. (2026). The theory of selection, optimization, and compensation. In M. Wang (Ed.), *The Oxford Handbook of Retirement* (p. 0). Oxford University Press. 10.1093/9780197699584.003.0007

[cit0019] Janlov, A. C. , Hallberg, I. R. , & Petersson, K. (2005). The experience of older people of entering into the phase of asking for public home help - a qualitative study. *International Journal of Social Welfare* , *14* (4), 326–336. 10.1111/j.1369-6866.2005.00375.x

[cit0020] Janlov, A. C. , Hallberg, I. R. , & Petersson, K. (2006). Older persons' experience of being assessed for and receiving public home help: do they have any influence over it? *Health & Social Care in the Community* , *14* (1), 26–36. 10.1111/j.1365-2524.2005.00594.x 16324185

[cit0021] Janssen, B. M. , Abma, T. A. , & Van Regenmortel, T. (2012). Maintaining mastery despite age related losses. The resilience narratives of two older women in need of long-term community care. *Journal of Aging Studies* , *26* (3), 343–354. 10.1016/j.jaging.2012.03.003

[cit0022] Jarling, A. , Rydström, I. , Ernsth-Bravell, M. , Nyström, M. , & Dalheim-Englund, A.-C. (2018). Becoming a guest in your own home: home care in Sweden from the perspective of older people with multimorbidities. *International Journal of Older People Nursing* , *13* (3). e12194. 10.1111/opn.12194 29603651

[cit0023] Jonsén, E. , Norberg, A. , & Lundman, B. (2015). Sense of meaning in life among the oldest old people living in a rural area in Northern. *Sweden. International Journal of Older People Nursing* , *10* (3), 221–229. 10.1111/opn.12077 25516075

[cit0024] Kierkegaard, S. (1996). Sjukdomen till döds: en kristlig psykologisk utveckling till uppbyggelse och uppväckelse [The disease unto death: a Christian psychological development for edification and revival]: Nimrod förlag AB.

[cit0025] Kirvalidze, M. , Hanson, E. , Magnusson, L. , Dahlberg, L. , Wimo, A. , Morin, L. , & Calderón-Larrañaga, A. (2025). The intensity of informal caregiving and its implications for older caregivers: a national survey in Sweden. *Scandinavian Journal of Public Health* , 14034948251335113. 10.1177/14034948251335113 PMC13323912 40312880

[cit0026] Lawton, M. P. , & Simon, B. (1968). The ecology of social relationships in housing for the elderly. Gerontologist (Washington, DC), *8* (2), 108–115. 10.1093/geront/8.2.108 5657480

[cit0027] Machielse, A. (2025). I’m a fighter and I do not give up’ – socially isolated older adults’ experiences with meaning in life. *Ageing and Society* , *45* (5), 879–900. 10.1017/S0144686X23000764

[cit0028] Maxwell, J. A. (2011). *A Realist Approach for Qualitative Research* . SAGE Publications Ltd.

[cit0029] Meinow, B. , Wastesson, J. W. , Kåreholt, I. , & Kelfve, S. (2020). Long-term care use during the last 2 years of life in Sweden: implications for policy to address increased population aging. Journal of the American Medical Directors Association, *21* (6), 799–805. 10.1016/j.jamda.2020.01.003 32081681

[cit0030] Meinow, B. , Goliath, I. , Wallcook, S. , Flink, M. , Siljehag, P. A. , Klinga, C. , Strehlenert, H. , & von Berens, Å. (2026). Older people in Sweden increasingly enter long-term care with extensive care needs–a register study of first-time users based on the SNAC Stockholm eldercare study. *European Journal of Ageing* , *23* , 7. 10.1007/s10433-025-00906-5 41528565 PMC12847603

[cit0031] Meleis, A. I. (2010). *Transitions theory: middle-range and situation-specific theories in nursing research and practice* . Springer Pub. Nwe York.

[cit0032] Menassa, M. , Stronks, K. , Khatmi, F. , Roa Díaz, Z. M. , Espinola, O. P. , Gamba, M. , Khatami, F. , Itodo, O. A. , Buttia, C. , Wehrli, F. , Minder, B. , Velarde, M. R. , & Franco, O. H. (2023). Concepts and definitions of healthy ageing: A systematic review and synthesis of theoretical models. *EClinicalMedicine* , *56* , 101821. 10.1016/j.eclinm.2022.101821 36684393 PMC9852292

[cit0033] Möllergren, G. (2026). Agency in later life: the unrecognised operatrices of a fragmented care system. Journal of Women & Aging. 1–19. 10.1080/08952841.2026.2643311 41848200

[cit0034] Möllergren, G. , Jönson, H. , & Granbom, M. (2025). Learning the trade of navigating and tinkering within a disparate welfare system: older adults as bricoleurs of home arrangements and support. Health & Place, *96* , 103562. 10.1016/j.healthplace.2025.103562 41076939

[cit0035] Näsman, Y. (2020). The theory of caritative caring: katie Eriksson's theory of caritative caring presented from a human science point of view. *Nursing Philosophy* , *21* (4), 1–10. 10.1111/nup.12321 32974999

[cit0036] Olsen, M. , Udo, C. , Dahlberg, L. , & Boström, A. M. (2022). Older Persons' views on important values in Swedish home care service: A semi-structured interview study. Journal of Multidisciplinary Healthcare, *15* , 967–977. 10.2147/JMDH.S347886 35535245 PMC9076494

[cit0037] Ottenvall Hammar, I. , Dahlin-Ivanoff, S. , Wilhelmson, K. , & Eklund, K. (2014). Shifting between self-governing and being governed: A qualitative study of older persons’ self-determination. *BMC Geriatrics* , *14* (1), 126. 10.1186/1471-2318-14-126 25432268 PMC4280698

[cit0038] Peterson, E. , & Brodin, H. (2022). Choice, needs or equality? Discursive struggles about defining home care for older people in Sweden. *Ageing and Society* , *42* (10), 2433–2453. 10.1017/S0144686X21000131

[cit0039] Philipp, R. , Walbaum, C. , Koch, U. , Oechsle, K. , Daniels, T. , Helmich, F. , Horn, M. , Junghans, J. , Kissane, D. , Lock, G. , Lo, C. , Mruk-Kahl, A. , Müller, V. , Reck, M. , Schilling, G. , Schulze, K. , von Felden, J. , Bokemeyer, C. , Härter, M. , & Vehling, S. (2025). Existential distress in advanced cancer: A cohort study. *General Hospital Psychiatry* , *94* , 184–191. 10.1016/j.genhosppsych.2025.02.023 40107200

[cit0040] Rocard, E. , & Llena-Nozal, A. (2022). Supporting informal carers of older people: Policies to leave no carer behind. (OECD Health Working Papers, No. 140). https://www.oecd.org/en/publications/supporting-informal-carers-of-older-people_0f0c0d52-en.html

[cit0041] Rostgaard, T. , & Szebehely, M. (2012). Changing policies, changing patterns of care: Danish and Swedish home care at the crossroads. *European Journal of Ageing* , *9* (2), 101–109. 10.1007/s10433-011-0209-1 28804411 PMC5547399

[cit0042] Schön, P. , & Heap, J. (2018). *ESPN European Social Policy Network thematic report on challenges in long-term care* . Sweden. Brussels: Directorate-General for Employment, Social Affairs and Inclusion Directorate C — Social Affairs.

[cit0043] Silpilä, J. (2019). *Social Care Services: The Key to the Scandinavian Welfare Model* . Routledge.

[cit0044] Silverglow, A. , Johansson, L. , Lidén, E. , & Wijk, H. (2022). Perceptions of providing safe care for frail older people at home: A qualitative study based on focus group interviews with home care staff. *SCANDINAVIAN JOURNAL OF CARING SCIENCES* , *36* (3), 852–862. 10.1111/scs.13027 34423863

[cit0045] Socialstyrelsen . (2012). *SOSFS (2012:3). Socialstyrelsens Allmänna Råd Om Värdegrunden i Social- Tjänstens Omsorg Om Äldre [The National Board of Health and Welfare’s General Advice on the Fundamental Values in the Social Services’ Care of Older People]* . Socialstyrelsen: [The National Board of Health and Welfare].

[cit0046] Socialstyrelsen . (2023). Äldreguiden - en tjänst från socialstyrelsen [The care guide for older adults - a service from The National Board of Health and Welfare]. Socialstyrelsen (National Board of Health and Welfare]. Available from: https://aldreguiden.se/mer-om-aldreomsorg/rattigheter/

[cit0047] Socialstyrelsen. ( 2024). Avgifter inom äldre- och funktionshindersomsorg [Fees for eldercare and care for people with disabilities]: Socialstyrelsen [National Board of Health and Welfare]; 2024 [Available from: https://www.socialstyrelsen.se/kunskapsstod-och-regler/omraden/aldre/regelverk/boende-och-stod-aldre/

[cit0048] Socialstyrelsen. ( 2026). Statistikdatabas för äldreomsorg [Statistics database for eldercare] ULR should be: https://sdb.socialstyrelsen.se/if_ald/

[cit0049] SOU 2020:19 . (2020). *God och nära vård - En reform för ett hållbart hälso- och sjukvårdssystem SOU 2020:19 [Good quality, local health care - A reform for a sustainable health care system]. In: Affairs] SMoS, editor* . Stockholm: Sveriges Riksdag [Swedish Parliament].

[cit0050] Statistikmyndigheten SCB. ( 2024). Statistikdatabasen, Folkmängden efter region, civilstånd, ålder och kön. År 1968 - 2023 [Statistical database, Population by region, marital status, age and sex. Year 1968 - 2023] Stockholm: Statistikmyndigheten SCB [Statistics Sweden]; 2024 [Available from: https://www.statistikdatabasen.scb.se/pxweb/sv/ssd/START__BE__BE0101__BE0101A/BefolkningNy/

[cit0051] Stockholms stad . (2009). Metodhandledning. En metodhandledning för biståndshandläggare inom äldreomsorgen I arbetet med kartläggning och bedömning av behov I den dagliga livsföringen, *[Method guide. A method guide for need assessors in eldercare in the work with mapping and assessment of needs in daily living]* . Äldreförvaltningen, Stockholms stad: [Eldercare administration, Stockholm municipality].

[cit0052] Stockholms stad. ( 2021). Riktlinjer för handläggning inom socialtjänstens äldreomsorg [Guidelines for the management of social services for older people]. Stockholm: Stockholms stad, Äldreförvaltningen [Department of ageing, The city of Stockholm].

[cit0053] Tong, A. , Sainsbury, P. , & Craig, J. (2007). Consolidated criteria for reporting qualitative research (COREQ): A 32-item checklist for interviews and focus groups. *International Journal for Quality in Health Care* , *19* (6), 349–357. 10.1093/intqhc/mzm042 17872937

[cit0054] Ueland, V. , Nåden, D. , & Lindström, U. (2018). Longing - a dynamic power in the becoming of health when suffering from cancer. Scandinavian Journal of Caring Sciences, *32* (2), 924–932. 10.1111/scs.12527 28940617

[cit0055] van Rhyn, B. , Barwick, A. , & Donelly, M. (2022). Embodied experiences and existential reflections of the oldest old. *Journal of Aging Studies* , *61* , 101028. 10.1016/j.jaging.2022.101028 35654552

[cit0056] Wacker, R. , & Roberto, K. (2016). Theories of help-seeking behavior: understanding community service use by older adults. In Bengtson, V. , Settersten, R. (Eds.), *Handbook of theories of ageing* . Springer publishing company. New York.

[cit0057] Wahl, H.-W. , Iwarsson, S. , & Oswald, F. (2012). Aging well and the environment: toward an integrative model and research agenda for the future. *The Gerontologist* , *52* (3), 306–316. 10.1093/geront/gnr154 22419248

[cit0058] Westerberg, K. , Hjelte, J. , & Josefsson, S. (2017). Understanding eldercare users' views on quality of care and strategies for dealing with problems in Swedish home help services. *Health & Social Care in the Community* , *25* (2), 621–629. 10.1111/hsc.12351 27109545

[cit0059] Wigzell O. (2020, 22 April). Pandemin visar att äldreomsorgen måste utvecklas [The pandemic highlights the need for development in eldercare]. *D* *agens Samhälle.* https://www.dagenssamhalle.se/opinion/debatt/pandemin-visar-att-aldreomsorgen-maste-utvecklas/

[cit0060] World Health Organization . (2017). *Integrated care for older people: guidelines on community-level interventions to manage declines in intrinsic capacity* . Geneva. 29608259

[cit0061] World Medical Association . (2024). *WMA Declaration of Helsinki – Ethical Principles for Medical Research Involving Human Participants* . World Medical Association. Available from: https://www.wma.net/policies-post/wma-declaration-of-helsinki/10.1001/jama.2024.21972 39425955

[cit0062] Wrzus, C. , Hänel, M. , Wagner, J. , & Neyer, F. J. (2013). Social network changes and life events across the life span: A meta-analysis. Psychological Bulletin (Washington, DC), *139* (1), 53–80. 10.1037/a0028601 22642230

[cit0063] Wulfse-Huisman, S. , Schuitemaker, P. , Veldhuizen, J. , Buurman-van Es, B. , Bleijenberg, N. , & Pel-Littel, R. (2025). Older adults' experiences of shared decision-making with district nurses on interventions to support independence: an interpretive phenomenological study. *Geriatric Nursing* , *65* , 103511. 10.1016/j.gerinurse.2025.103511 40639074

