# Supplementary material for: Coming to terms with the need for home care: a reflexive thematic analysis of older adults’ experiences in Sweden
Source: Int J Qual Stud Health Well-being. 2026 Jul 24;21(1):2707705. doi: 10.1080/17482631.2026.2707705 (PMC13403359; doi:10.1080/17482631.2026.2707705)
Supplement: Revised_Supplementary_material_2.docx [file ZQHW_A_2707705_SM3261.docx]

**Title**

Coming to terms with the need for home care: A reflexive thematic analysis of older adults’ experiences in Sweden.

**Journal name**

International Journal of Qualitative Studies on Health and Well-being

**Author names, affiliations and e-mail addresses**

P Alencar Siljehag ^1, 2^, pernilla.alencarsiljehag@aldrecentrum.se

Å von Berens ^1, 2^, asa.vonberens@aldrecentrum.se

B Meinow ^1, 2^, bettina.meinow@aldrecentrum.se

A Liljas ^2, 3^, ann.liljas@ki.se

J Agerholm ^2^, janne.agerholm@ki.se

^1^ Stockholm Gerontology Research Center, Stockholm, Sweden

^2^ Aging Research Center, Karolinska Institutet, Stockholm University, Stockholm, Sweden

^3.^ Department of Global Public Health, Karolinska Institutet, Stockholm, Sweden

**Supplementary material 2** Consent form

**Project: Home care for the first time**

*Consent to participate in the project*

I have received verbal and written information and had the opportunity to ask questions about the studies into being granted and receiving home care for the first time. I can keep the written information and have been informed that participation is voluntary and that I can withdraw without any consequences.

- I agree to participate in the project “Home care for the first time”
- I agree that the information I share in this project will be stored n accordance with the instructions in “Information to research participants”

| Place and date | Signature |
| --- | --- |
|  |  |
|  | Name clarification |
|  |  |
