# Supplementary material for: Coming to terms with the need for home care: a reflexive thematic analysis of older adults’ experiences in Sweden
Source: Int J Qual Stud Health Well-being. 2026 Jul 24;21(1):2707705. doi: 10.1080/17482631.2026.2707705 (PMC13403359; doi:10.1080/17482631.2026.2707705)
Supplement: Revised_Supplementary_material_3.docx [file ZQHW_A_2707705_SM3250.docx]

**Title**

Coming to terms with the need for home care: A reflexive thematic analysis of older adults’ experiences in Sweden.

**Journal name**

International Journal of Qualitative Studies on Health and Well-being

**Author names, affiliations and e-mail addresses**

P Alencar Siljehag ^1, 2^, pernilla.alencarsiljehag@aldrecentrum.se

Å von Berens ^1, 2^, asa.vonberens@aldrecentrum.se

B Meinow ^1, 2^, bettina.meinow@aldrecentrum.se

A Liljas ^2, 3^, ann.liljas@ki.se

J Agerholm ^2^, janne.agerholm@ki.se

^1^ Stockholm Gerontology Research Center, Stockholm, Sweden

^2^ Aging Research Center, Karolinska Institutet, Stockholm University, Stockholm, Sweden

^3.^ Department of Global Public Health, Karolinska Institutet, Stockholm, Sweden

**Supplementary material 3** Full interview guide

| Semi-structured questions |
| --- |
|  |
| What does a typical day look like for you? |
| What was your living situation and health like before applying for home care? |
| What considerations did you make before applying for home care? |
| How did you find the application process? |
| How did you feel about receiving home care for the first time? |
| Do the services you have been granted correspond to your perceived needs? |
| Do other people (family, neighbors, etc.) provide care and support in addition to home care services? Has this changed in any way recently? |
| How do you perceive the collaboration between different actors (e.g., home care/primary care or home care/hospital care) in connection with the start of home care? |
|  |
