# Supplementary material for: Coming to terms with the need for home care: a reflexive thematic analysis of older adults’ experiences in Sweden
Source: Int J Qual Stud Health Well-being. 2026 Jul 24;21(1):2707705. doi: 10.1080/17482631.2026.2707705 (PMC13403359; doi:10.1080/17482631.2026.2707705)
Supplement: Revised_Supplementary_material_4.docx [file ZQHW_A_2707705_SM3245.docx]

**Title**

Coming to terms with the need for home care: A reflexive thematic analysis of older adults’ experiences in Sweden.

**Author information**

P Alencar Siljehag ^1, 2^, Å von Berens ^1, 2^, B Meinow ^1, 2^, A Liljas ^2, 3^, J Agerholm ^2^

^1^ Stockholm Gerontology Research Center, Stockholm, Sweden

^2^ Aging Research Center, Karolinska Institutet, Stockholm University, Stockholm, Sweden

^3.^ Department of Global Public Health, Karolinska Institutet, Stockholm, Sweden

**Address of corresponding author**

Pernilla Alencar Siljehag Institutional address: Stiftelsen Stockholms läns Äldrecentrum, Sveavägen 155, 113 46 Stockholm, Sweden. Phone: +46-8-690 5868. E-mail: pernilla.alencarsiljehag@aldrecentrum.se

**Supplementary material 4** Full list of codes

| **Grasping the gap** | **Desiring continuity in identity** | **Cherishing trustful relationships** | **Conducting a dialogue** |
| --- | --- | --- | --- |
| Codes | Codes | Codes | Codes |
| Afraid of falling | Self-reliant self-image | Pity for my loved ones | Re-evaluate independence |
| Eventually a habit | Tough being the weakest | Hard on loved ones | Accept to avoid dwelling |
| Stay in good spirits | Resistance to help | Like an intruder | Tune in and reflect |
| Grasp new needs | Falling short of norm | My children think I need help |  |
| It's limbing along | Will I be myself again? |  |  |
| Afraid of the loneliness | Ill while feeling fine |  |  |
| It came like a bang |  |  |  |
| The overwhelming responsibility | |  |  |
| What will become of me? |  |  |  |
| I don’t like being this helpless |  |  |  |
| Trust in professional competence | |  |  |
| Relearned what used to feel obvious | |  |  |
| Increasingly difficult to manage | |  |  |
| Scared of my own shortcomings | |  |  |
| We did everything together |  |  |  |
| At times tired from responsibility | |  |  |
| Now understanding the feeling of offense | |  |  |
| Hesitant toward unfamiliar |  |  |  |
| Missing lost places and habits |  |  |  |
| Lonely in a cut back life |  |  |  |
| Trapped by fear and responsibility | |  |  |
| My best time was then |  |  |  |
