# Supplementary material for: Coming to terms with the need for home care: a reflexive thematic analysis of older adults’ experiences in Sweden
Source: Int J Qual Stud Health Well-being. 2026 Jul 24;21(1):2707705. doi: 10.1080/17482631.2026.2707705 (PMC13403359; doi:10.1080/17482631.2026.2707705)
Supplement: Revised_Supplementary_material_1_.docx [file ZQHW_A_2707705_SM3268.docx]

**Title**

Coming to terms with the need for home care: A reflexive thematic analysis of older adults’ experiences in Sweden.

**Journal name**

International Journal of Qualitative Studies on Health and Well-being

**Author names, affiliations and e-mail addresses**

P Alencar Siljehag ^1, 2^, pernilla.alencarsiljehag@aldrecentrum.se

Å von Berens ^1, 2^, asa.vonberens@aldrecentrum.se

B Meinow ^1, 2^, bettina.meinow@aldrecentrum.se

A Liljas ^2, 3^, ann.liljas@ki.se

J Agerholm ^2^, janne.agerholm@ki.se

^1^ Stockholm Gerontology Research Center, Stockholm, Sweden

^2^ Aging Research Center, Karolinska Institutet, Stockholm University, Stockholm, Sweden

^3.^ Department of Global Public Health, Karolinska Institutet, Stockholm, Sweden

**Supplementary material 1** Participant information

**Project: Home care for the first time**

*Information to research participants*

We would like to ask if you would like to participate in a research project. This document provides information about the project and what participation entails.

*What is the project about and why are you asking me to participate?*

During the last decades, the number of beds in municipal round-the-clock care and in hospitals has fallen dramatically, while eldercare provided in ordinary homes has been expanded to a corresponding extent. People who use home care tend to be older and suffer from a greater burden of disease today than they did 20 years ago. Knowledge about people’s living situations when they receive eldercare for the first time is sparse. The purpose of this project is to increase knowledge about the characteristics of the life situation of older people who are granted eldercare for the first time and to explore their experiences of how the services granted correspond with their needs and expectations.

We have received information from needs assessors in your municipality or your local pensioners' association that you may be interested in sharing your experiences of being 65 or older and recently receiving home care. We would therefore like to ask if you are interested in participating in this study.

The principal research organization is Karolinska Institutet. The principal research organization refers to the organization responsible for the project. The project application has been approved by the Swedish Ethical Review Authority, reference number for their review is 2023-06089-01.

How does the project work?

You participate in an interview where you freely answer questions about your experiences of applying for and receiving home care. You will also be asked to provide background information such as your age, gender, and education. The interview will last approximately 45 minutes and will be audio recorded. It will be carried out approximately one month after you have received your decision on home care allowance. The interview will take place at a physical or digital meeting, depending on your preferences. A relative or friend may be present. You can influence the time and place.

*Possible consequences and risks of participation*

No negative consequences are expected from participation.

*What happens to my data?*

The data will be handled in such a way that unauthorized people do not have access to it. Data collected within the project will be reported in de-identified form and in a way that makes it impossible to trace it back to you. Data will be stored in accordance with EU data protection regulations and Karolinska Institutet is the data controller for the project. According to EU data protection regulations, you have the right to access the information about you that is used in the project and, if necessary, have any errors corrected. You may also request that information about you be deleted and that the use of your personal data be restricted. If you would like to access the data, please contact Janne Agerholm, Karolinska institutet, janne.agerholm@ki.se. The data protection officer can be contacted by e-mail at dataskyddsombud@ki.se. If you are not satisfied with how your data is being handled, you have the right to complain to the Data Protection Authority which is the supervisory authority.

*How can I access the project?*

If you have any questions about the individual data collected in the project, please contact the person responsible as indicated below. The project results may be presented at educational conferences and in scientific publications. You have the option to decline to receive the results. If unexpected health findings are discovered, such as some form of mental illness during the interview, the researchers will refer you to care and support.

*Compensation*

You will not receive any compensation for participating in this project.

*Participation is voluntary*

Your participation is voluntary, and you may choose to discontinue at any time. If you choose not to participate or wish to withdraw your participation, you do not need to explain why, and it will not affect your future care or treatment.

**Responsible for the project are:**

Pernilla Alencar Siljehag (pernilla.siljehag@ki.se)

PhD student at the Stockholm Gerontology Research Center and Aging Research Center, Department of Neurobiology, Care Sciences and Society, Karolinska Institutet

Janne Agerholm (janne.agerholm@ki.se)

Assistant professor at Aging Research Center, Department of Neurobiology, Care Sciences and Society, Karolinska Institutet
